# A Cross-Sectional Study of Health-Related Quality of Life in Patients with Predominantly Antibody Deficiency

**DOI:** 10.21203/rs.3.rs-4612913/v1

**Published:** 2024-07-18

**Authors:** Ahmed Elmoursi, Baijun Zhou, Mei-Sing Ong, Joseph S. Hong, Andrew Pak, Megha Tandon, Natalia Sutherland, Daniel V. DiGiacomo, Jocelyn R. Farmer, Sara Barmettler

**Affiliations:** Massachusetts General Hospital; Massachusetts General Hospital; Harvard Medical School, Harvard Pilgrim Health Care Institute; Massachusetts General Hospital; Massachusetts General Hospital; Massachusetts General Hospital; Massachusetts General Hospital; Hackensack Meridian School of Medicine; Beth Israel Lahey Health; Massachusetts General Hospital

**Keywords:** Predominantly antibody deficiency, Health-related quality of life, CDC HRQoL-14, Behavioral risk factor surveillance system, Immunodeficiency, Common variable immunodeficiency, Hypogammaglobulinemia, Immunoglobulin therapy, Chronic disease management, Mental health, Physical health, Patient-reported outcomes

## Abstract

Health-related quality of life (HRQoL) measures individual well-being across physical, psychological, and social domains. Patients with predominantly antibody deficiency (PAD) are at risk for morbidity and mortality, however, the effect of these complications on HRQoL requires additional study. Patients with PAD were asked to voluntarily complete the Centers for Disease Control (CDC) HRQoL-14 Healthy Days Measure questionnaire. These results were compared to data from the CDC-initiated Behavioral Risk Factor Surveillance System (BRFSS), a cross-sectional questionnaire including questions from CDC-HRQOL-14. Statistical analyses included two-proportion Z-test, t-tests, and analysis of variance. 83 patients with PAD completed the survey. Patients were sub-stratified into mild (23.7%), moderate (35.5%), severe (40.8%), and secondary (8.4%) PAD. “Fair or poor” health status was reported in 52.6% of PAD patients. Mental health challenges ≥ 14 days/month occurred in 25% of patients. Physical health issues ≥ 14 days/month was reported in 44.7% of patients. Activity limitations were noted by 80.3% of patients. There were no statistically significant differences by PAD severity. Patients with autoinflammatory disease co-morbidities reported more mental health challenges compared to those without (78% vs. 54.3%, p = 0.02). Compared to the CDC-BRFSS data, significantly more patients with PAD reported “fair or poor” health status (53% vs 12.0%; p < 0.0001), mental health challenges (24.1% vs 14.7%; p = 0.02), and poor physical health (44.6% vs 8.0%; p < 0.0001). Patients with PAD had significantly reduced HRQoL compared to CDC-BRFSS respondents from a similar geographical region. Decreased HRQoL was prevalent across all PAD severity levels. Additional research is needed to improve HRQoL for patients with PAD.

## INTRODUCTION

Predominantly antibody deficiency (PAD) is the most frequently diagnosed inborn error of immunity (IEI) and the most common primary immunodeficiency disorder worldwide.(1–5) Patients with PAD are susceptible to recurrent infections due to reduced antibody levels and/or inadequate vaccination responses.(6, 7) However, the impact of PAD extends beyond recurrent infections; affected individuals often face a spectrum of comorbidities, including autoimmune diseases and lymphoproliferative disorders, which can contribute to progressive end-organ damage and a diminished life expectancy.(3)

The concept of health-related quality of life (HRQoL) is becoming increasingly recognized as an important metric for evaluating patient well-being and for guiding therapeutic choices in both clinical and research settings.(8) HRQoL captures a comprehensive, multifaceted evaluation of an individual’s physical, psychological, emotional, and social functioning. This in turn reflects a wide-ranging and complex domain of interest among diverse stakeholders including healthcare providers, researchers, policymakers, and families.(8–10) Increasing evidence positions HRQoL as an essential indicator for assessing treatment effectiveness and the overall health of those with chronic conditions. This approach extends beyond traditional clinical measures such as survival, adverse events, and death rates to offer a holistic view of the effects of disease and treatment on a patient’s life from their own perspective.(11, 12)

Studies have shown that HRQoL measurements, when tailored to specific conditions, provide a more precise evaluation of the impacts of medical interventions on patient lives when compared to general clinical assessments. (13) This approach facilitates the identification of various health determinants and guides the development of personalized treatment, management, and preventive strategies, aligning with healthcare’s ultimate goal of enhancing QoL while also prolonging lives. (14)

The principal strategy for both prophylaxis and management of PAD is the administration of immunoglobulin G (IgG) replacement, either intravenously (IVIG) or subcutaneously (SCIG). This therapeutic approach has proven to be both effective and safe in reducing the incidence and severity of infections in PAD patients.(15) Despite this, patients continue to face ongoing challenges, such as recurrent infections, autoimmune and lymphoproliferative complications, and the need for frequent acute care visits. These challenges are attributed to the chronic nature of PAD, treatment side effects, and the complexities of their care management. This limits patients’ social and physical activities (16, 17), contributing to an increased risk of fatigue (18), depression (19), anxiety (20), cardiovascular diseases, cancer, sleep disorders, and mental health conditions (21–23), which may severely affect their psychological and physical health, and consequently, reduce their overall quality of life (QoL).

Despite the progress in treatment modalities that have improved survival rates for individuals with PAD, the impact of PAD on HRQoL has not been thoroughly researched. Therefore, we sought to assess HRQoL in PAD by utilizing the Centers for Disease Control (CDC) Health-Related Quality of Life (HRQoL)-14 Healthy Days Measure questionnaire (24), and to compare the HRQoL of PAD patients with that of the general population, utilizing data from the CDC’s Behavioral Risk Factor Surveillance System (BRFSS).(25, 26)

## METHODS

This cross-sectional observational study was designed to assess the HRQoL in patients diagnosed with PAD. Adult PAD patients (age ≥ 18 years) were consented at Mass General Brigham (MGB). Participants voluntarily completed the Centers for Disease Control (CDC) HRQoL-14 Healthy Days Measure questionnaire (24) during routine visits or at intermittent time points. This was conducted under an Institutional Review Board (IRB) approved protocol (#2018P002713) between September 24, 2019, and February 12, 2024. The diagnoses of PAD were confirmed through manual chart review by a clinical immunologist, and met consensus definitions.(6, 27, 28)

A total of 83 patients with PAD were included. HRQoL-14 Healthy Days Measure is a self-report questionnaire designed to assess HRQoL among adult participants. It contains 14 items distributed across two primary dimensions: Mental Health (MH) (4 items), and Physical Health (PH) (10 items). Some items are assessed on a categorical scale and others require respondents to provide a continuous measure, such as reporting the number of unhealthy days they have experienced within the past 30 days. Taken together, these measures indicate a patient’s health status with a higher count suggesting a poorer health condition.

Supplementary sections delve into specifics such as activity limitations and health symptoms such as pain, depression, anxiety, sleep disorders, and energy levels. It also inquires about respondents’ recent health conditions and any limitations to their activities. The completion of the questionnaire typically requires no more than five minutes. The total and dimensional scores are interpreted directly, with no need for transformation to a percentage of a maximum possible score. For items with missing responses, the CDC provides guidelines on how to handle incomplete data based on the specific requirements of the analysis.(24) The questionnaire was originally developed in English and is publicly available for use.

Patients with PAD were subclassified as mild (Primary hypogammaglobulinemia, IgG subclass deficiency, and specific antibody deficiency [SAD]), moderate (uncomplicated common variable immunodeficiency [CVID]), and severe (complicated PAD defined as a presence of co-occurring autoinflammatory clinical features (28)), as previously published.(29, 30) Patients with confounding variables at the time of immunodeficiency diagnosis (e.g., clonal lymphocyte population or ongoing immunosuppression without the potential for discontinuation) were considered to be secondary PAD. We compared the responses across the three severity levels among primary PAD patients, and further we compared responses from PAD patients with data from the CDC-initiated Behavioral Risk Factor Surveillance System (BRFSS), which included HRQOL-14 responses from a control comparator group of the general population in the Boston area, consisting of 801,582 respondents, in 2021.(25, 26)

Data were collected on patient demographics including age, gender, race, and specific PAD diagnoses. Information regarding clinical characteristics and disease severity was gathered through patient medical records. Descriptive statistics were used to summarize the sample characteristics, with mean values described for continuous variables and frequencies and percentages for categorical variables. We used a two-proportion Z-test to compare the responses from PAD patients in our study with the CDC-BRFSS data. Chi-square tests and analyses of variance (ANOVA) were used to compare the responses across three severity levels among PAD patients. Statistical analyses were completed with SAS 9.4 (SAS Institute, Cary, NC) and Prism Version 7.01 (Reston, VA). A two-tailed p-value of < 0.05 was considered significant.

## RESULTS

### PAD patient demographics

This cross-sectional observational study evaluated 83 adult patients diagnosed with PAD using the CDC HRQoL-14 Healthy Days Measure questionnaire. Among these patients, 91.6% (n = 76) had primary PAD and 8.4% (n = 7) had secondary PAD. The average age of participants was 54.8 years, with a standard deviation (SD) of 15.4 years. 71% were female and 97.6% were non-Hispanic White. The most common type of PAD was complicated Common Variable Immunodeficiency (CVID) (e.g. CVID with autoinflammatory features or a more severe immunophenotype), observed in 53% of patients (n = 44), followed by uncomplicated CVID, which constituted 24.1% (n = 20) of the cases. Primary hypogammaglobulinemia was present in 8.4% (n = 7) of participants, secondary hypogammaglobulinemia in 8.4% (n = 7), specific antibody deficiency accounted for in 3.6% (n = 3), and IgG subclass deficiency accounted for 2.4% (n = 2) of the PAD types within our study population (Table 1). Primary PAD patients were stratified by their disease severity; 23.7% (n = 18) had mild PAD, 35.5% (n = 27) had moderate PAD, and 40.8% (n = 31) had severe PAD. This sample was representative of our overall MGB cohort of patients with PAD in terms of age, race/ethnicity, and disease distribution (Table S1).

### HRQoL in PAD patients

We compared the HRQoL in adult patients with primary PAD, stratified by disease severity. Our analysis compared core and individual HRQoL-14 questions across mild, moderate, and severe PAD patient groups (Table 2). Core HRQoL questions evaluated patients’ perceptions of their overall health and the extent to which they experienced periods of suboptimal mental or physical health lasting more than two weeks.

**Figure 1:**
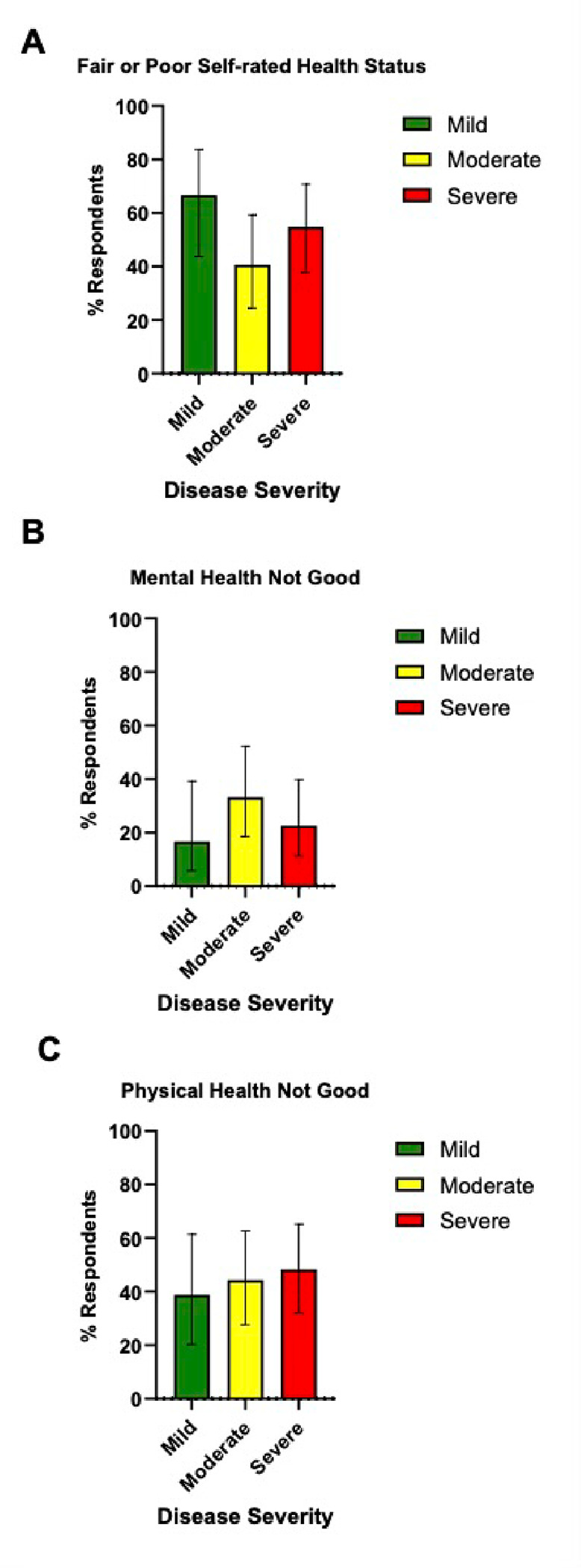
Percentage of poor self-rated health status, mental health, and physical health among primary PAD patients by clinical severity. The figure represents the distribution of self-rated health status: A) Fair or poor self-rated health status, B) Mental health not good for ≥14 days, and C) Physical health not good for ≥14 days, segregated by disease severity. The vertical axis indicates the percentage of respondents, and the horizontal axis classifies the severity of PAD into mild (green bar; n=18), moderate (yellow bar; n=27), and severe (red bar; n=31) PAD adult patients. Each bar indicates the proportion of individuals who reported their health status. Error bars represent the 95% confidence interval of the proportion of respondents. Although a trend of increased poor health status with greater disease severity is observed, the differences did not reach statistical significance.

The assessment of fair or poor self-rated health status revealed a gradient across disease severity groups. 66.7% of mild PAD patients (n = 18) reported “fair or poor” health status, compared to 40.7% of those with moderate PAD (n = 27), and 54.8% in the severe PAD group (n = 31) (Fig. 1A). Mental health challenges lasting for 14 or more days were reported by 16.7% of mild PAD patients, compared to 33.3% of patients with moderate PAD and 22.6% of those with severe PAD (Fig. 1B). The evaluation of physical health revealed that 38.9% of mild PAD patients (n = 7) experienced poor physical health for 14 or more days, a slight increase to 44.4% for moderate PAD patients (n = 12), and nearly half, 48.4%, for severe PAD patients (n = 15), reported prolonged periods of poor physical health (Fig. 1C). There was no statistically significant difference across the disease severity groups.

The impact of PAD on patients’ daily activities was profound across all severity groups. The majority of patients reported limitations in activities due to health problems with 88.9% in the mild PAD group (n = 16), 92.0% in the moderate PAD group (n = 23), and 71.0% in the severe PAD group (n = 22). Patients across all severity groups reported similar mean numbers of days with poor physical health (13.4 for mild vs. 13.1 for moderate vs. 13.6 for severe PAD) and mental health (5.5 for mild vs. 10.6 for moderate vs. 8.0 for severe PAD) within the past 30 days. The long-term impact on daily activities, as measured by the number of months that activities were limited by health issues, varied widely with patients with mild PAD reporting a mean of 32.0 months, patients with moderate PAD reporting a mean of 111.5 months, and patients with severe PAD reporting a mean of 52.2 months.

Pain was reported as a key factor limiting usual activities for an average of 11.3, 9.4, and 9.0 days in the mild, moderate, and severe PAD groups, respectively. The percentage of patients needing assistance with personal care varied across severity groups, with 5.6% of mild PAD patients, 14.8% of moderate PAD patients, and 13.3% of severe PAD patients reporting this need. Assistance with routine needs was consistently reported by patients across severity levels, with 50.0% in the mild (n = 9), 51.9% in the moderate (n = 14), and 36.7% in the severe (n = 11) groups.

Emotional well-being, measured by days felt sad or depressed, was variable between groups, with a slightly higher mean of 9.5 days in the moderate PAD group compared to 4.3 and 4.8 days in the mild and severe groups, respectively. Similarly, anxiety levels were similar across severity groups, with the average number of days feeling anxious being 8.9 for mild, 12.6 for moderate, and 7.0 for severe PAD. Sleep disturbances, measured by the number of days not feeling rested or having enough sleep, also showed no significant difference, with an average of 14.9 days for mild, 15.2 for moderate, and 11.4 for severe PAD patients.

**Figure 2:**
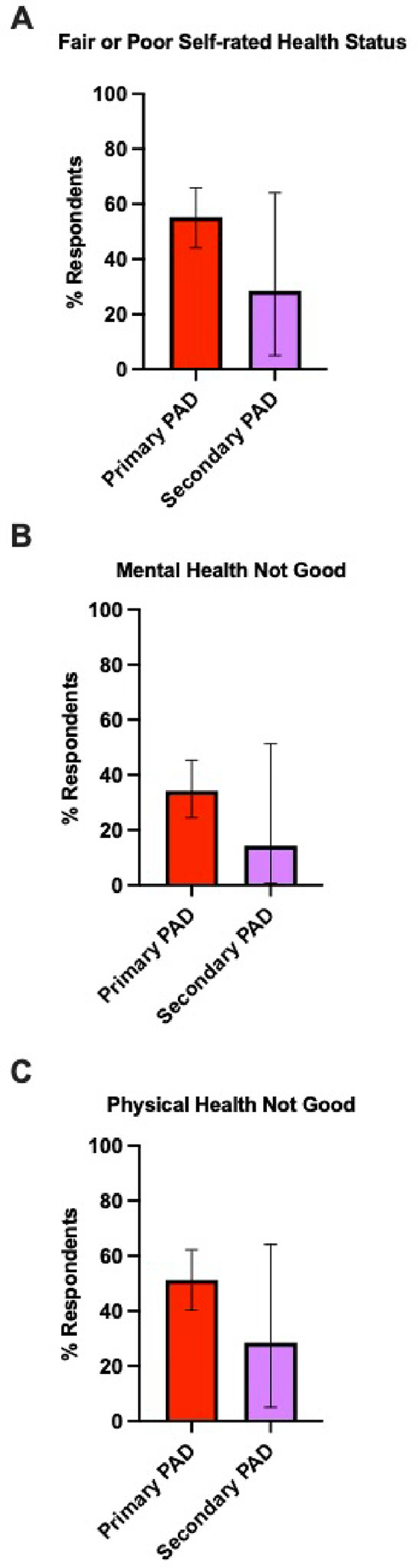
Percentage of poor self-rated health status, mental health, and physical health among primary PAD patients compared to secondary PAD. The figure represents the distribution of self-rated health status: A) Fair or poor self-rated health status, B) Mental health not good for ≥14 days, and C) Physical health not good for ≥14 days, compared between adult patients with primary PAD (red bar; n=76) and secondary PAD (purple bar; n=7). Each bar indicates the proportion of individuals who reported their health status. Error bars represent the 95% confidence interval of the proportion of respondents. Although primary PAD patients show a trend of worse health status compared to secondary PAD patients, the differences did not achieve statistical significance.

We compared the core HRQoL-14 questions between primary PAD patients (n = 76) and secondary PAD patients (n = 7). 55.2% of primary PAD patients reported “fair or poor” health status, compared to 28.5% of secondary PAD patients (p = 0.18) (Fig. 2A). In the mental health domain, 34.2% of primary PAD patients reported challenges lasting 14 or more days, whereas only 14.3% of secondary PAD patients reported similar challenges (p = 0.28) (Fig. 2B). Physical health assessments revealed that 51.3% of primary PAD patients experienced poor physical health for 14 or more days, compared to 28.5% of secondary PAD patients (p = 0.25) (Fig. 2C).

**Figure 3:**
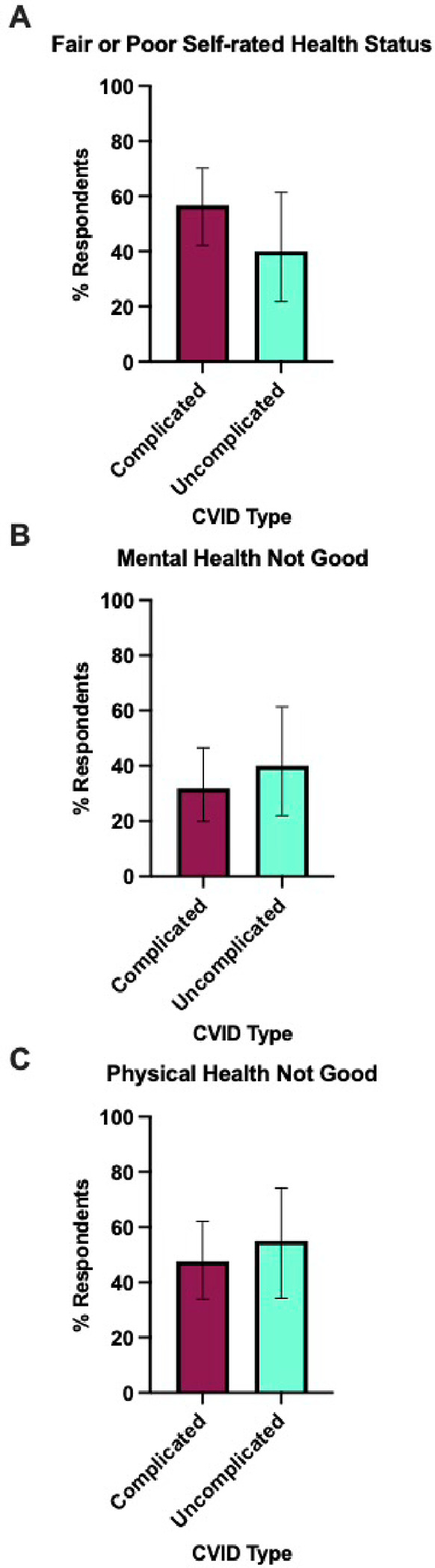
Percentage of poor self-rated health status, mental health, and physical health among CVID patients by complication status. The figure represents the distribution of self-rated health status: A) Fair or poor self-rated health status, B) Mental health not good for ≥14 days, and C) Physical health not good for ≥14 days, compared between complicated (maroon bar; n=44) and uncomplicated (cyan bar; n=20) CVID patients. Each bar indicates the proportion of individuals who reported their health status. Error bars represent the 95% confidence interval of the proportion of respondents.

Among CVID patients, we compared the core HRQoL-14 responses between complicated (n = 44) and uncomplicated (n = 20) CVID patients. Patients with complicated CVID were more likely to rate their health as “fair” or “poor” (56.8%, n = 25) compared to those with uncomplicated CVID (40%, n = 8) (p = 0.21) (Fig. 3A). Mental health challenges lasting for 14 or more days were reported by 31.8% of patients with complicated CVID, compared to a slightly higher 40% of those with uncomplicated CVID (p = 0.52) (Fig. 3B). The evaluation of physical health revealed that 47.7% of patients with complicated CVID experienced poor physical health for 14 or more days, compared to a prolonged period reported by 55% of those with uncomplicated CVID (p = 0.59) (Fig. 3C).

Although trends in the data suggest a relationship between increased disease severity and poorer HRQoL—especially in terms of physical health and fair or poor self-rated health status—the differences did not reach statistical significance, likely due to sample size. Similarly, when we stratified the analyses by therapies received and genetic testing, no statistically significant differences were observed when comparing those with and without immunoglobulin replacement therapy (IgRT) (Supplemental Figure S1), having received immunomodulator therapy (Supplemental Figure S2), or having an identified genetic etiology (Supplemental Figures S3). This may imply that the diagnosis of PAD itself, irrespective of the severity level, imposes a substantial burden on the HRQoL of patients.

**Figure 4:**
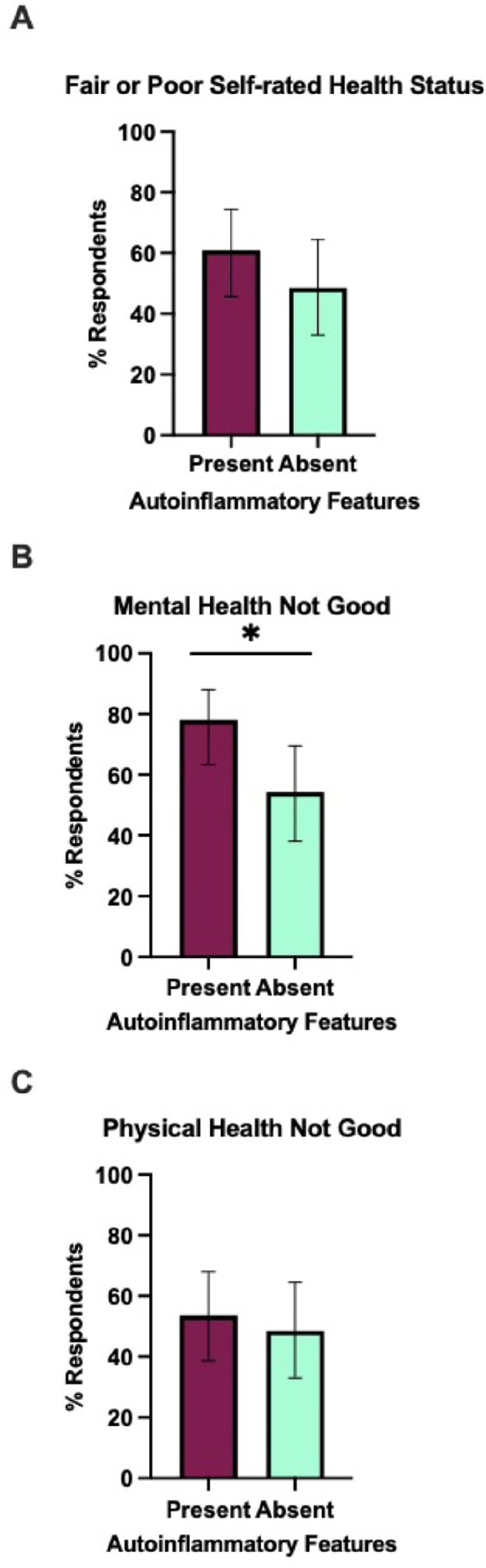
Percentage of poor self-rated health status, mental health, and physical health among primary PAD patients by autoinflammatory features. The figure represents the distribution of self-rated health status: A) Fair or poor self-rated health status, B) Mental health not good for ≥14 days, and C) Physical health not good for ≥14 days, segregated by presence of autoinflammatory features. The vertical axis indicates the percentage of respondents, and the horizontal axis differentiates between PAD patients with (maroon bar; n=41) and without (cyan bar; n=35) autoinflammatory features. Each bar shows the proportion of individuals reporting their health status. Error bars indicate the 95% confidence interval of the proportion of respondents. A significant difference in mental health status is noted (*p<0.05), with worse mental health observed in patients with present autoinflammatory features.

We further evaluated the core HRQoL-14 responses between primary PAD patients with (n = 41) and without (n = 35) autoinflammatory features. In the mental health domain, 78% of patients with autoinflammatory features reported challenges lasting 14 or more days, significantly higher than the 54.3% of patients without these features (p = 0.02) (Fig. 4B). A similar trend was noted in the other two domains. Patients with autoinflammatory features were more likely to rate their health as “fair” or “poor” (61%) compared to those without autoinflammatory features (48.6%) (p = 0.28) (Fig. 4A). Physical health assessments indicated that 53.7% of patients with autoinflammatory features experienced poor physical health for 14 or more days, compared to 48.6% of those without (p = 0.66) (Fig. 4C). However, these differences were not statistically significant.

### HRQoL is lower in PAD patients compared to healthy controls

**Figure 5:**
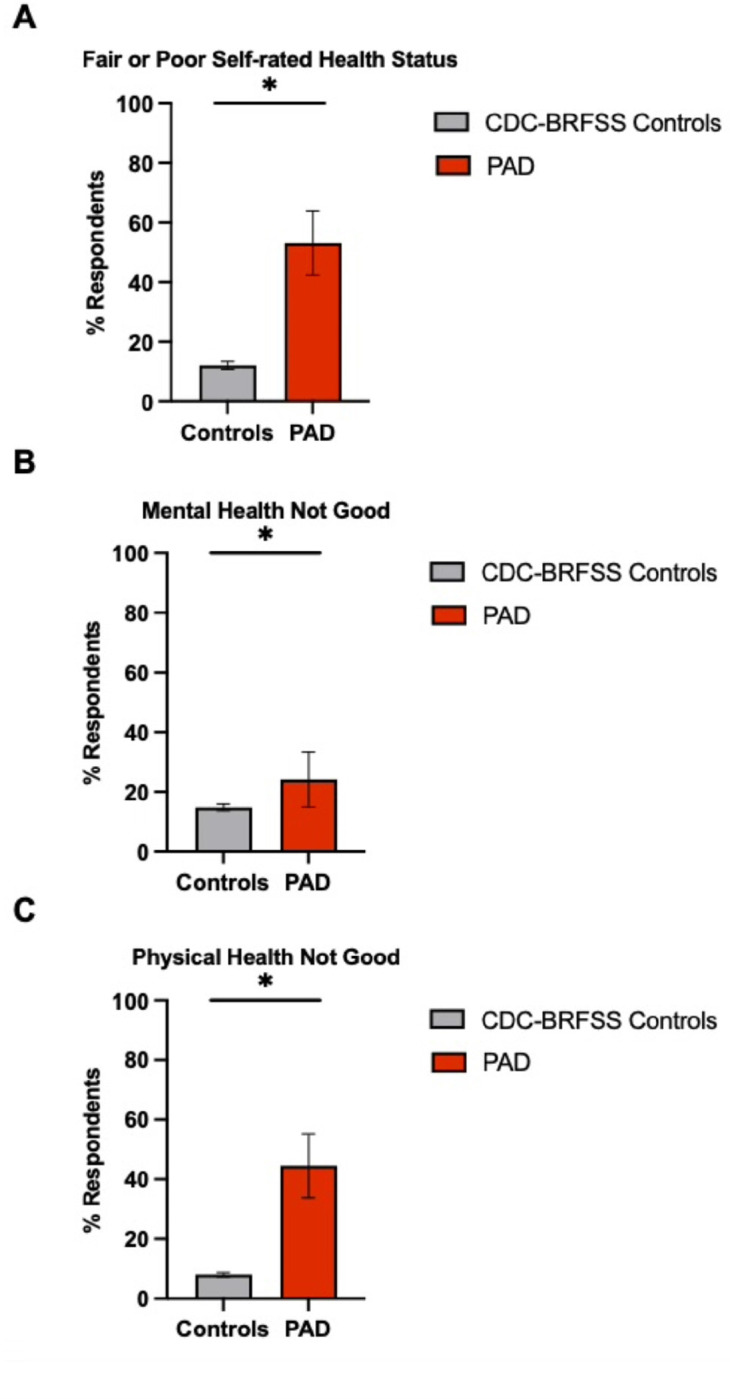
Percentage of poor self-rated health status, mental health, and physical health among PAD patients compared to CDC-BRFSS data controls. The figure represents the distribution of self-rated health status: A) Fair or poor self-rated health status, B) Mental health not good for ≥14 days, and C) Physical health not good for ≥14 days, compared between adult patients with PAD (red bar; n=83) and control data derived from the CDC’s BRFSS data surveying the general population (gray bar; n=801,582). Each bar indicates the proportion of individuals who reported their health status. HC stands for healthy controls from CDC-BRFSS data. Error bars represent the 95% confidence interval of the proportion of respondents. *p<0.05. The results demonstrate that PAD patients have significantly lower HRQoL across all measured domains compared to the control group.

We conducted a comparative analysis of HRQoL-14 Healthy Days Measure questionnaire responses between PAD patients (n = 83) and a control group of the general population (n = 801,582) from CDC-BRFSS data from a similar geographic area (Table 3). Patients with PAD were more likely to evaluate their health as “fair” or “poor” (53%, n = 44), which was significantly higher than the 12.0% in the CDC-BRFSS comparator group (n = 96,190), indicating a marked impact of PAD on self-perceived health (p < 0.0001) (Fig. 5A). In the mental health domain, 24.1% of PAD patients (n = 20) reported challenges lasting 14 or more days, which was significantly more than the 14.7% in the CDC-BRFSS comparator group (n = 117,833) (p = 0.02) (Fig. 5B). Physical health assessments further illustrated the PAD patients’ burden, with 44.6% (n = 37) experiencing poor physical health for 14 or more days, notably higher than the 8.0% in the CDC-BRFSS comparator group (n = 64,127) (p < 0.0001) (Fig. 5C).

## DISCUSSION

In our study, we evaluated the HRQoL using the CDC HRQoL-14 Healthy Days Measure questionnaire among patients with mild, moderate, and severe primary PAD. Additionally, we compared HRQoL across several strata including primary and secondary PAD, complicated and uncomplicated CVID patients, presence and absence of autoinflammatory features, and between those receiving or not receiving immunoglobulin replacement and immunomodulator therapies. We also compared the HRQoL of PAD patients with that of the general population, utilizing data from the CDC-BRFSS. Consistent with prior research, we observed that PAD has a significant negative impact on patients’ quality of life when compared to the general population.

Our assessment of HRQoL among PAD patients revealed that the burden of the disease on QoL was evident across all levels of disease severity. Our analysis did not reveal a direct correlation between increasing severity and lower HRQoL scores; instead, it revealed a more complex pattern. While a high percentage of mild PAD patients reported fair or poor self-rated health status, this did not linearly increase with disease severity. Despite nearly half of the severe PAD group reporting extended periods of poor physical health, this percentage was not significantly higher than that reported by the mild group. Activity limitations, another key indicator of HRQoL, were reported by a substantial majority of patients across all severity groups. Moreover, the need for assistance with personal care and routine needs, as well as emotional and sleep disturbances, showed no significant variation across the disease severity spectrum.

Comparing primary and secondary PAD patients, we found that primary PAD patients reported worse HRQoL outcomes. A higher proportion of primary PAD patients rated their health as “fair or poor” and experienced prolonged periods of poor physical and mental health compared to secondary PAD patients.

Notably, primary PAD patients with autoinflammatory features exhibited significantly worse mental health outcomes, with 78% reporting challenges lasting 14 or more days, compared to 54.29% of those without autoinflammatory features. While trends in physical health and overall health ratings followed a similar pattern, these differences were not statistically significant, which may have been limited by sample size. Similarly, our extended analysis, including stratifications based on immunoglobulin replacement therapy, immunomodulator therapy, and identified genetic etiology, did not reveal statistically significant differences across the three domains. These findings suggest that the diagnosis of PAD itself, regardless of severity, places a substantial burden on patients’ HRQoL. This indicates that factors beyond disease severity, such as psychosocial or environmental factors, psychological resilience, and coping mechanisms developed over time, may significantly influence quality of life. These factors could potentially buffer the impact of disease progression on perceived well-being, highlighting the need for comprehensive care approaches that address not only the physical but also the mental and social aspects of living with PAD.

The comparison of HRQoL between PAD patients and the comparator population from the BRFSS data further highlights the significant impact of PAD on patients’ lives. PAD patients were more than four times as likely to report fair or poor self-rated health compared to the CDC-BRFSS comparator group, a difference that was highly significant. PAD also had a substantial negative impact on mental health, with nearly a quarter reporting poor mental health for two weeks or more, a rate significantly higher than in the BRFSS data. Similarly, the physical domain was severely affected, with PAD patients experiencing poor physical health for extended periods at a rate more than five times higher than that of the general population. These findings highlight the need for targeted interventions in PAD patients, designed to mitigate the extensive impact of both mental and physical heath domains, and address the broad and complex needs that extend beyond the clinical diagnosis of PAD.

While treatments such as immunoglobulin replacement therapy (IgRT), antibiotic prophylaxis, and in severe cases, hematopoietic stem cell transplantation (HSCT), have notably improved survival rates for patients with PAD, patients still continue to face challenges in their physical and mental health. Therefore, assessing the HRQoL is critical for understanding the comprehensive impact of PAD on patients’ daily lives and overall well-being. Of note, here we did not see QoL benefit from immunomodulator therapy. These data may suggest that additional or alternative targeted approaches are needed. Future work is needed to investigate these outcomes by specific immunomodulator therapy type, duration, and targeted treatment approach.

While several studies have investigated HRQoL in adults and pediatric patients with IEI, research has yet to focus on adult patients with PAD or to draw comparisons with healthy controls. Generally, data from these studies indicate that IEI patients have a significantly poorer overall health status and self-rated health when compared with those suffering from other chronic diseases.(16) Research reveals that IEI patients experience more severe mental health issues, such as depression, isolation, and anxiety, than those observed in the general population.(14) These findings are consistent with previous research that has established psychosocial factors as substantial determinants of the incidence, QoL, and mortality rates associated with chronic illnesses.(31, 32)

There were several limitations to our study. Although we assessed a considerable number of PAD patients, the cohort was predominantly female and non-Hispanic White. Thus, the external validity of our findings might be limited, highlighting the need for future research involving PAD patients of more diverse backgrounds who are matched to healthy controls.(33, 34) Furthermore, the small sample size and the cross-sectional design of the study may restrict the generalizability of our findings and our ability to establish causality. The reliance on self-reported measures, while insightful for understanding patient experiences, could introduce response biases. Additionally, the recruitment of participants from a single center might not accurately represent the wider PAD patient population. Despite these limitations, our findings provide an essential foundation for future research aimed at enhancing the QoL for individuals with PAD. Further studies with larger, more diverse populations and longitudinal designs are needed to clarify the long-term impacts of PAD on HRQoL and validate the interventions that may improve outcomes for these patients.

In conclusion, this study provides insights into the significant impact of PAD on HRQoL across all severity levels, demonstrating the necessity for care strategies that include mental and social health support alongside medical management. The findings also stress the importance of interventions tailored to all severity levels, not limited to those classified as a clinically severe phenotype. Though progress has been achieved in treating PAD, there is a heightened need to focus on improving patients’ health-related quality of life. This requires creating strong methods for evaluating HRQoL and meeting the psychological and social needs of those with PAD. Future research, with a focus on long-term studies and a broader, more inclusive, demographic is needed to develop effective interventions that enhance quality of life for all patients with PAD.

## Figures and Tables

**Table 1:** Patient demographics

**Predominantly antibody deficiency, n**	**83**
**Average age (years, [std])**	54.8 (15.4)
**Sex**	
Male (% [n])	28.9 (24)
Female (% [n])	71.1 (59)
**Race (% [n])**	
Non-Hispanic White	97.6 (81)
Black/African American	0 (0)
Asian	1.2 (1)
American Indian/Alaska	0 (0)
Native Hawaiian Other Pacific Islander	0 (0)
Declined/Two or more/Other/Unknown	1.2 (1)
**Predominantly antibody deficiency type (% [n])**	
Complicated CVID	53.0 (44)
Common variable immunodeficiency (CVID)	24.1 (20)
Primary Hypogammaglobulinemia	8.4 (7)
Secondary Hypogammaglobulinemia	8.4 (7)
Specific antibody deficiency	3.6 (3)
IgG subclass deficiency	2.4 (2)

**Table 2:** Comparison of HRQoL-14 responses among mild, moderate, and severe PAD patients.

	Mild PAD (n = 18)	Moderate PAD (n = 27)	Severe PAD (n = 31)	P-value
**Core questions**				
Fair or poor self-rated health status (% [n])	66.7 (12)	40.7 (11)	54.8 (17)	0.22
Mental health not good for ≥ 14 days (% [n])	16.7 (3)	33.3 (9)	22.6 (7)	0.41
Physical health not good for ≥ 14 days (% [n])	38.9 (7)	44.4 (12)	48.4 (15)	0.81
**Individual questions**				
Activity limitation due to health problem^a^ (% [n])	88.9 (16)	92.0 (23)	71.0 (22)	0.11
Need for personal care assistance^b^ (% [n])	5.6 (1)	14.8 (4)	13.3 (4)	0.74
Need for assistance with routine needs^c^ (% [n])	50.0 (9)	51.9 (14)	36.7 (11)	0.47
Number of days during the past 30 days with poor physical health^d^ (mean [std])	13.4 (11.3)	13.1 (10.2)	13.6 (11.5)	0.98
Number of days during the past 30 days with poor mental health^e^ (mean [std])	5.5 (8.2)	10.6 (10.4)	8.0 (8.8)	0.19
Number of days during the past 30 days that poor physical or mental health affected usual activities^f^ (mean [std])	9.7 (10.4)	11.4 (9.9)	9.0 (9.2)	0.64
Number of months that activities limited by health problem (mean [std])	32.0 (34.2)	111.5 (380.0)	52.2 (79.5)	0.48
Number of days during the past 30 days that pain impacted usual activities (e.g., self-care, work, or recreation (mean [std])	11.3 (11.2)	9.4 (9.3)	9.0 (10.6)	0.74
Number of days during the past 30 days felt depressed^g^ (mean [std])	4.3 (8.2)	9.5 (9.3)	4.8 (7.9)	0.06
Number of days during the past 30 days felt anxious or tense^h^ (mean [std])	8.9 (10.9)	12.6 (10.8)	7.0 (8.0)	0.10
Number of days during the past 30 days lacked sufficient rest or sleep^i^ (mean [std])	14.9 (11.0)	15.2 (9.9)	11.4 (11.1)	0.34
Number of days during the past 30 days felt health and energetic^j^ (mean [std])	6.1 (8.9)	5.5 (7.4)	10.0 (11.0)	0.16

a Are you LIMITED in any way in any activities because of any impairment or health problem?

b Because of any impairment or health problem, do you need the help of other persons with your PERSONAL CARE needs, such as eating, bathing, dressing, or getting around the house?

c Because of any impairment or health problem, do you need the help of other persons in handling your ROUTINE needs, such as everyday household chores, doing necessary business, shopping, or getting around for other purposes?

d Now thinking about your physical health, which includes physical illness and injury, for how many days during the past 30 days was your physical health not good?

e Now thinking about your mental health, which includes stress, depression, and problems with emotions, for how many days during the past 30 days was your mental health not good?

f During the past 30 days, for about how many days did poor health keep you from doing your usual activities, such as self-care, work, or recreation?

g During the past 30 days, for about how many days have you felt SAD, BLUE, or DEPRESSED?

h During the past 30 days, for about how many days have you felt WORRIED, TENSE, or ANXIOUS?

i During the past 30 days, for about how many days have you felt you did NOT get ENOUGH REST or SLEEP?

j During the past 30 days, for about how many days have you felt VERY HEALTHY AND FULL OF ENERGY?

**Table 3:** Comparison of HRQoL-14 responses among PAD patients and BRFSS respondents.

	Sample Size	Cases No.	Proportion (95% CI)	P-value
**Fair or poor self-rated health status (% [n])**				
PAD	83	44	53.0% (42.3%–63.8%)	<0.0001
CDC-BRFSS Controls	801,582	96,190	12.0% (10.7%–13.5%)	
**Mental health not good for ≥14 days (% [n])**				
PAD	83	20	24.1% (14.9%–33.3%)	0.02
CDC-BRFSS Controls	801,582	117,833	14.7% (13.6%–15.9%)	
**Physical health not good for ≥14 days (% [n])**				
PAD	83	37	44.6% (33.9%–55.3%)	<0.000
CDC-BRFSS Controls	801,582	64,127	8.0% (7.3%–8.7%)	

## Abbreviations

PAD: predominantly antibody deficiency
HRQoL: health–related quality of life
CDC: centers for disease control and prevention
BRFSS: behavioral risk factor surveillance system
IVIG: intravenous immunoglobulin
SCIG: subcutaneous immunoglobulin
CVID: common variable immunodeficiency
SAD: specific antibody deficiency
IgG: immunoglobulin G
IRB: institutional review board
HSCT: hematopoietic stem cell transplantation
IgRT: immunoglobulin replacement therapy
ANOVA: analysis of variance
